# E-nergEYEze, a vision-specific eHealth intervention based on cognitive behavioral therapy and self-management to reduce fatigue in adults with visual impairment: study protocol for a randomized controlled trial

**DOI:** 10.1186/s13063-021-05935-w

**Published:** 2021-12-28

**Authors:** Manon H. J. Veldman, Hilde P. A. van der Aa, Christina Bode, Hans Knoop, Carel T. J. Hulshof, Marc Koopmanschap, Edwin Stavleu, Ger H. M. B. van Rens, Ruth M. A. van Nispen

**Affiliations:** 1 Amsterdam UMC, Vrije Universiteit Amsterdam, Ophthalmology, Amsterdam Public Health Research Institute, De Boelelaan, 1117 Amsterdam, The Netherlands; 2 Department of Psychology, Health and Technology, University of Twente, Enschede, The Netherlands; 3 Amsterdam UMC, University of Amsterdam, Medical Psychology, Amsterdam Public Health Research Institute, Meibergdreef 9, Amsterdam, The Netherlands; 4 Amsterdam UMC, University of Amsterdam, Coronel Institute of Occupational Health, Amsterdam Public Health Research Institute, Meibergdreef 9, Amsterdam, The Netherlands; 5 Institute of Health Policy and Management, Erasmus University, Rotterdam, The Netherlands; 6 Royal Dutch Visio, Centre of Expertise for Visually Impaired and Blind People, Huizen, The Netherlands; 7 Elkerliek Hospital, Ophthalmology, Helmond, The Netherlands

**Keywords:** Fatigue, Visual impairment, Cognitive behavioral therapy, Self-management, eHealth, Randomized controlled trial

## Abstract

**Background:**

More than half of the adults with visual impairment experience severe symptoms of fatigue, with a negative impact on daily life. Since there is no evidence-based treatment to reduce fatigue in adults with visual impairment, we developed E-nergEYEze, an eHealth intervention based on cognitive behavioral therapy and self-management tailored to the needs of visually impaired adults. The aim is to describe the study protocol of a randomized controlled trial testing E-nergEYEze.

**Methods:**

A randomized controlled trial will be conducted to investigate the cost-effectiveness and cost-utility of E-nergEYEze to reduce fatigue severity compared to care as usual from a healthcare and societal perspective. A total of 172 severely fatigued adults with visual impairment will be recruited and randomized to either the E-nergEYEze intervention plus care as usual or to care as usual only (ratio 1:1). Inclusion criteria are having a visual impairment, experiencing severe fatigue (Checklist Individual Strength – subscale Fatigue Severity: CIS-FS > 35), being 18 years or older, understanding the Dutch language, and having access to the internet. The intervention consists of one face-to-face session and a computer training followed by internet-based modules with information and assignments on coping with fatigue. During this 5-month intervention, participants will be digitally supported by a social worker. All measurements will be administered at baseline, after 6 and 12 months, and additionally, those related to cost-effectiveness at 3 and 9 months. The primary outcome is fatigue severity (CIS-FS).

**Discussion:**

Severe fatigue on top of visual impairment compromises quality of life and is associated with incremental societal costs that largely determine the economic burden of low vision or blindness. E-nergEYEze contributes to the evidence base of potentially feasible interventions to reduce the important health-related consequences of vision loss and could fulfill the gap in knowledge, skills and treatment options for low vision services.

**Trial registration:**

Dutch Trial Register NTR7764. Registered on 28 May 2019.

**Supplementary Information:**

The online version contains supplementary material available at 10.1186/s13063-021-05935-w.

## Administrative information

**Table:** 

Title {1}	E-nergEYEze, a vision-specific eHealth intervention based on cognitive behavioral therapy and self-management to reduce fatigue in adults with visual impairment: study protocol for a randomized controlled trial.
Trial registration {2a and 2b}.	Dutch Trial Register identifier: NTR7764. Registry name: Reducing fatigue in visually impaired adults with the E-nergEYEze intervention. Date registered: 28-05-2019. Date enrollment first participant: 06-10-2020
Protocol version {3}	Date**:** 04-03-2020. Version**:** 7
Funding {4}	This study was externally funded and supported by the Dutch Organization for Health Research and Development (“ZonMw program Inzicht”, grant No 60-00635-98-219). ZonMW has no role in the design and conduct of the study or in the writing of the manuscript and will not have any role during its execution, analyses, interpretation of the data, or decision to submit results.
Author details {5a}	1. Amsterdam UMC, Vrije Universiteit Amsterdam, Ophthalmology, Amsterdam Public Health research institute, De Boelelaan 1117, Amsterdam, The Netherlands 2. Department of Psychology, Health and Technology, University of Twente, Enschede, The Netherlands 3. Amsterdam UMC, University of Amsterdam, Medical Psychology, Amsterdam Public Health research institute, Meibergdreef 9, Amsterdam, The Netherlands 4. Amsterdam UMC, University of Amsterdam, Coronel Institute of Occupational Health, Amsterdam Public Health research institute, Meibergdreef 9, Amsterdam, The Netherlands 5. Institute of Health Policy and Management, Erasmus University, Rotterdam, The Netherlands 6. Royal Dutch Visio, Centre of Expertise for Visually Impaired and Blind People, The Netherlands 7. Elkerliek Hospital, Ophthalmology, Helmond, The Netherlands **Corresponding author** Full name**:** Manon Hubertina Jozephine Veldman Postal address**:** Amsterdam UMC Dept. Ophthalmology, PK KTC 4-023 attn. M. Veldman PO Box 7057 1007 MB Amsterdam, the Netherlands Email**:** m.veldman@amsterdamumc.nl
Name and contact information for the trial sponsor {5b}	Sponsor contact information Trial sponsor**:** Amsterdam University Medical Centres Contact Name**:** dr. R.M.A. van Nispen Address**:** De Boelelaan 1118, 1081 HZ Amsterdam Telephone: + 31(0)20-4444795 Email**:** r.vannispen@amsterdamumc.nl.
Role of sponsor {5c}	The sponsor has no role in the design and conduct of the study or in the writing of the manuscript and will not have any role during its execution, analyses, interpretation of the data, or decision to submit results.
Composition, roles and responsibilities of the coordinating centre, steering committee, endpoint adjudication committee, data management team {5d}	The research team of the coordinating centre is composed of researchers of the Low Vision Research group, and provide the day to day support of the trial involving: the design, conduct, data analyses, data interpretation and submission of results of the study. The project team, composed of professionals in the field (e.g. ophthalmologists, behavioral scientists, social workers, researchers) and patient representatives, share insights and experiences; they will be involved in making decisions and obtain an advisory role. The project team meet 3 times over the course of the trial.

## Introduction

### Background

Fatigue is a significant problem in adults with visual impairment that needs to be addressed urgently [1]. Severe fatigue in persons with visual impairment seems to be related to vision loss; they experience fatigue more often compared to persons with normal sight [2–4]. A large cross-sectional study showed that severe fatigue experienced by persons with visual impairment (*n*=247) is associated with depression, a lower quality of life, less self-efficacy, and unsuccessful coping strategies compared to persons with normal sight [5, 6]. In addition, work stressors such as problems with work pace and travel to work are associated with fatigue [7]. Severely fatigued adults with visual impairment describe fatigue as a mental and physical sensation, with feelings of languidness, heaviness, and inertia. Some experienced fatigue as an uncontrollable and unpredictable sensation that is overwhelming and sudden. Fatigue seems to be caused by specific factors that are (in)directly associated with vision loss, such as a high cognitive load to process and memorize information, the effort needed to establish visual perception, and negative cognitions. The impact of fatigue on emotional and cognitive functioning, social roles, and participation have been reported as problematic by severely fatigued adults with visual impairment [6]. Consequences of fatigue include difficulty maintaining energy to endure daily activities, difficulty concentrating, and crossing one’s personal boundaries regarding energy balance [6, 8]. Quantitative insight showed that persons with visual impairment can use several strategies to reduce fatigue, such as physical exercise, acceptance of fatigue, undertaking social activities, limiting visual perception, and balancing activities with periods of relaxation [6].

Understanding fatigue, perceiving it as more controllable, and experiencing less emotional consequences are examples of positive views about fatigue that are closely related to the reduction of fatigue severity [9]. Cognitive behavioral therapy (CBT) [10–14], self-management (SM) [13, 15–18], and exercise [14, 19] have shown to reduce fatigue in people with cancer, rheumatoid arthritis, polyarthritis, multiple sclerosis (MS) or diabetes. Guided eHealth interventions have proven to be successful in these populations as well [11, 12, 15–17, 20]. A recent review suggested that such interventions can be most effective when they are tailored to specific symptoms, such as fatigue, and adapted for specific populations [21]. Those interventions were designed to deal with disease-specific perpetuating factors of fatigue in web-based modules and were offered through an internet portal [11, 12, 15]. Additionally, eHealth interventions can be considered to improve access to healthcare and reduce healthcare costs because they are independent of time and place, require less effort from professionals then face-to-face interventions, and stimulate patient empowerment [22].

Adults with visual impairment have indicated that reducing fatigue is a rehabilitation goal of high importance [8] and the widespread societal burden of fatigue in this population warrants prioritization of future resources to reduce and manage its impact on the individual level and society as a whole [1]. Although eHealth has been investigated in patients with retinal exudative diseases who receive anti-VEGF treatment to reduce depression and/or anxiety [22], to date, there is no evidence of a structural and effective online treatment available addressing factors to reduce vision-related fatigue in adults with visual impairment.

Combining knowledge obtained in previous studies [1, 5–7, 11, 12, 15, 23], we developed E-nergEYEze, a blended vision-specific eHealth intervention, based on cognitive behavioral therapy and self-management, to reduce severe fatigue in adults with visual impairment. Here we present a protocol of a single-blind randomized controlled trial (RCT) to investigate the cost-effectiveness and cost-utility of E-nergEYEze in reducing severe fatigue in adults with visual impairment, compared with care as usual from a healthcare and societal perspective.

## Methods

### Study design

In this RCT we investigate the effectiveness of an eHealth intervention aimed at reducing fatigue severity in adults with visual impairment. A single-blind pragmatic RCT will be conducted comparing two parallel arms in which patients are stratified based on employment status and low vision service region.

### Sample size

Group sample sizes of 64 per trial arm achieve 80,15% power to reject the null hypothesis of equal means, when the population mean difference is 4.0 on the Checklist Individual Strength – subscale Fatigue Severity (CIS-FS) with a standard deviation for both groups of 8.0 and with an alpha of 0.05 (two-sided) [10, 19, 24–26]. Since we expect a dropout after 12 months of 25%, approximately 86 visually impaired patients are required per trial arm (*N* = 172 in total). In a former fatigue study [1], approximately 25% of participants had a paid job and in a previous trial unequal distributions were found in both trial arms [27]. Therefore, randomization will be stratified by employment status as well as by low vision service region represented by a mental healthcare professional to equally distribute numbers of participations per region.

### Recruitment

Approximately 2000 patients, (formerly) registered at low vision service organizations in the Netherlands (Royal Dutch Visio and Bartiméus), will be invited by these services to participate in this trial by sending them large-print study information. Recruitment will take place in three waves, between August 2020 and April 2022 approximately, or until the total number of study participants has been achieved. Potential participants may indicate their interest to participate in the trial by filling out and sending a form with contact information to the research team in Amsterdam UMC. Subsequently, the research team will contact them by telephone to answer questions and inform the patient about the informed consent procedure. When patients are interested to participate in the trial, the research team will send the consent form by regular mail with a return envelope. Following enrollment by the research team, patients will be screened for eligibility (Table 1) after which the baseline assessment and randomization will take place (Table 2).

**Table 1:** Inclusion and exclusion criteria

Inclusion criteria	
➤ Being visually impaired according to WHO criteria [28, 29] of any cause (ophthalmic disorder) ➤ Being 18 years or older ➤ Understanding of the Dutch language ➤ Experiencing severe fatigue (CIS-FS^a^-score > 35) [30] ➤ Having access to the internet	
Exclusion criteria	
➤ Experiencing severe cognitive limitations assessed with the 6-item screener (short validated MMSE^b^) [31] ➤ Currently receiving treatment, or having received treatment in the last 12 months by a medical specialist for a comorbid disease that clearly is the main cause of fatigue (MS, cancer, psychiatric disorder).	

^a^Checklist Individual Strength – subscale Fatigue Severity

^b^Mini-Mental State Examination

**Table 2:** Study design

		Study period					
	Enrolment	Baseline assessment	Allocation	Post-allocation assessments			
Timepoint	*T -1*	*T 0*		*T 1*	*T 2*	*T 3*	*T 4*
Enrolment:							
Informed consent	X						
Eligibility screening	X						
Baseline assessment		X					
Randomization			X				
Intervention:							
*E-nergEYEze*		X		X	X	X	X
*Care as usual*		X		X	X	X	X
Assessments:							
*General questionnaire*		X					
*Primary outcome*	X				X		X
*Secundary outcomes*							
*- Clinical effectiveness*		X		X	X	X	X^a^
*- Cost effectiveness*		X^b^		X	X^b^	X	X^b^
*Process evaluation*		X			X^c^		
*Working mechanism*		X			X		X

^a^Additional assessment “Past life Events”

^b^Additional assessment “Euroqol-5 Dimensions with 5 levels”

^c^Additional assessment for intervention group “Dutch Mental Healthcare thermometer”

### Randomization

All patients who provide written informed consent for participation and who meet the inclusion criteria will be individually randomized by the research team. Information on employment status and low vision service region is obtained during the informed consent procedure and baseline assessment. Participants will be allocated to the intervention or control group (Table 2). Randomization will be performed according to a 1:1 ratio using random sequence block randomization (blocks of 2, 4, and 6), taking into account the two stratification parameters. Due to the nature of the intervention participants cannot be masked. Allocation to the intervention or control group will be communicated with the participant by email by an unmasked researcher. At the start of the trial and during telephone contacts, participants are asked not to disclose the nature of their treatment allocation during the follow-up assessments. Masked research assistants will conduct telephone interviews during the follow-up assessments. The research team will regularly check whether a research assistant is still masked by asking to guess to which trial arm a participant is allocated.

### Intervention

E-nergEYEze is based on CBT and SM, a targeted psychotherapy with a practical approach to encourage the change of dysfunctional thinking and behavioral patterns to deal with a medical condition and reduce symptoms. The intervention has been developed by (mental) health professionals (i.e., social workers and psychologists), patients and researchers. The content of the intervention is inspired by existing modules of guided eHealth CBT treatment [11, 23], but were adapted to the predisposing factors and determinants of fatigue for our specific population [1, 5–7] and by taking into account interventions found in literature [10–20]. Its development and subsequent usability and feasibility studies will be published in a separate manuscript. The content consists of an introduction and eight thematic internet-based modules, of which two modules are optional, guided by social workers and information and communication technology (ICT) trainers from Royal Dutch Visio and Bartiméus (two large low vision services organizations in the Netherlands). The modules inform the participant on how to cope with fatigue, focusing on vision-specific aspects of fatigue and fatigue-related beliefs and behaviors.

All participants will be assigned to a social worker for one face-to-face session to discuss the purpose of the intervention and to set personal goals. Additionally, they will receive a computer training given by an ICT trainer with instructions on software features. Both sessions will be scheduled within a month after baseline assessment to commence the intervention. Participants can log in to the online portal with their email address and their own, unique password. The participant will start the E-nergEYEze intervention with an introduction module on understanding vision-related fatigue. The successive eight modules respond to the perpetuating factors of fatigue with information and assignments, meant to change dysfunctional beliefs and fatigue maintaining behavioral patterns (Table 3). Participants can follow the intervention at home or at any other preferred place during a period of 5 months.

**Table 3:** Overview of the modules of the E-nergEYEze intervention

Module titles and content of E-nergEYEze intervention	
Module title	Content and assignments;
Introduction Understanding vision-related fatigue	Psycho-education on visual impairment and its association with fatigue, including perpetuating factors of chronic fatigue.
Module 1 Dealing with a visual impairment	Emotional impact of vision loss, concerning processing feelings of grief and coping strategies.
Module 2 Replacing dysfunctional fatigue-related thoughts	Understanding the effect of beliefs on fatigue and fatigue maintaining behavior, exercises to formulate helping beliefs, and shifting attention away from fatigue.
Module 3 Graded activity program	Practical exercises to distribute activities in a balanced way e.g. for patients with a boom-bust activity pattern, followed by a graded activity program.
Module 4 Communication and social support	Understanding how verbal and non-verbal communication has a role in the association between visual impairment and fatigue, but also exercises to express independence from others, changes in social relationships, and being assertive.
Module 5 Relaxation	Recognizing experienced stress and learning how to release tension by relaxation exercises.
Module 6 Improving sleep	Understanding the importance of a fixed sleep-wake cycle, obtaining a personalized optimal sleep-wake cycle, and useful strategies to sleep or stay awake.
Module 7 Work optimization	Balancing work-related stressors and energy sources, exercises to improve self-efficacy and work resumption
Module 8 The future	Letting go the “rules” of E-nergEYEze, achieving personal goals for the future, recognizing signs of relapse, and experiencing healthy levels of fatigue

The intervention will be guided by trained and supervised social workers. They will receive an email notification on finished assignments at the end of each module to provide feedback. Feedback will be given digitally within one week to answer questions and motivate participants to continue the intervention. If it is noticed that a participant does not adhere to the treatment program, the social worker will try to improve adherence. Telephone contact will be offered during the training for a short evaluation or if necessary for the progress or safety of the participant. The intensity of contact will depend on the participant’s need. Social workers will be supervised by psychologists. The professionals will document all contact moments in addition to the digital registration of the online platform. Theoretically, it is possible that the intervention could worsen symptoms of fatigue induced by greater awareness of fatigue-related symptoms and an emotional response can be evoked by the content. This will be monitored by the social worker and reported to the research team as a study-related (serious) adverse event ((S)AE). Equal to the control group receiving care as usual, the intervention group is allowed to receive any other care that is needed, from within or outside the low vision services.

Social workers, ICT trainers, and psychologists will receive a one-day course in good clinical practice, software features, and delivering digital feedback. During the trial, peer-to-peer coaching will be organized to evaluate the process, to coach each other, and to discuss further improvements. This pragmatic design focuses on real-life practice conditions.

### Care as usual

The intervention and control group will both receive care as usual, which includes any health care utilization that is necessary to maintain the health of a participant, such as care from a general practitioner, hospital care, or care provided by low vision service organizations. The control group is an active comparator.

### Outcome measures

Assessment of both groups will take place at baseline (T0), after 6 (T2), and 12 (T4) months, and additionally at 3 (T1) and 9 (T3) months for questionnaires related to the cost-effectiveness to avoid recall bias. All assessments will be conducted by telephone, in which standardized validated questionnaires will be administered. Baseline assessment (T0) will take place before randomization and includes collecting demographic data and data on information about comorbid conditions. Subsequent assessments will be conducted by structured interviews with validated questionnaires by masked research assistants, who will immediately enter participant responses into Castor (data entry software) [32]. Assessment T0, T2 and T4 will take approximately 1 h, T1 and T3 will take 15 min. Participants are offered the opportunity to perform any assessment in two separate sessions on different days to accommodate their needs. An overview of all measurement instruments and properties is provided in Additional file 1.

#### Primary outcome

The primary outcome is fatigue severity and will be assessed as a part of the Checklist Individual Strength (CIS), the Fatigue Severity (FS) subscale [30]. The CIS consists of 20 items addressing fatigue, concentration, motivation, and activities, with a 7-point Likert scale. The subscale FS consists of eight items, ranging from 8 to 57 points, and a cut-off score of 35 points or higher indicating severe fatigue. The CIS-FS scale is considered a valid and reliable tool to assess fatigue severity [33] and has previously been used to assess fatigue as an effect of interventions for other chronic diseases [10–12, 14].

#### Secondary outcomes

Secondary outcomes measure (1) the impact of fatigue on daily life with the Modified Fatigue Impact Scale (MFIS) [34]; (2) adaptation to vision loss and acceptance of the disability in relation to oneself or to others with the 9-item Adaptation to Vision Loss (AVL) [35, 36]; (3) symptoms of depression with the 9-item Patient Health Questionnaire (PHQ-9) [37]; (4) symptoms of anxiety with the Hospital Anxiety Depression scale – Anxiety subscale (HADS-A) [38]; (5) vision-related quality of life with the Impact of Visual Impairment questionnaire (IVI) [39, 40]; (6) work functioning (WRFQ 2.0) [41] and work participation (Work Ability Index 1 item) [42]; (7) perception and assessment of work with the Need for Recovery questionnaire (NFR) [43]; and (8) circadian rhythm sleep disorders, insomnia and hypersomnia with the Holland Sleep Disorders Questionnaire (HSDQ) [44] at baseline, 6, and 12 months. Life events that happened in the past year will be measured at 12 months with the Past Life Events (PLE) questionnaire [45].

#### Cost evaluation outcomes

To assess cost-effectiveness, (1) health care utilization will be assessed with the Medical Costs Questionnaire (iMCQ) and the costs of the intervention will be valued using the standard costs per unit for health care utilization with the Dutch costs manuals (guideline at https://www.zorginstituutnederland.nl/); (2) medication use, valued by using pricing from Dutch Medical costs guidelines (www.medicijnkosten.nl); (3) absenteeism and presenteeism from paid and unpaid work with the iMTA Productivity Cost Questionnaire (iPCQ) [46]. Productivity losses will be valued using the friction cost approach, assuming that after a certain period of time (i.e., 161 days) the sick employee is replaced. Therefore, lost productivity costs are generated only during the friction period [47]; (4) health-related quality of life with the Euroqol-5 Dimensions with 5 levels (EQ-5D-5L), which is the preferred measure to determine quality-adjusted life-years in economic evaluations [48].

#### Process evaluation outcomes

A process evaluation using six parameters will be performed among participants in the intervention group and social workers to investigate fidelity and uptake of the intervention: (1) Competencies of Cognitive Therapy Scale-Self Report (CCTS-SR) at baseline and 6 months [49]; (2) compliance will be operationalized in the intervention group at T2 by participants rating their effort and social workers rating the participants’ compliance to the E-nergEYEze intervention, based on a 10-point scale (0 = no effort/compliance to 10 = full effort/compliance); (3) the intervention platform Minddistrict (https://www.minddistrict.com/en-gb) logs how often and for how long the intervention has been used; (4) recall is operationalized after each module by social workers rating the degree to which participants seem to remember the last modules on a 10-point scale (0 = participant remembers nothing to 10 = participant remembers everything); (5) Dutch Mental Healthcare (MH) thermometer of satisfaction [50]; and (6) therapist satisfaction and adherence will be assessed by means of evaluation forms filled out by social workers during the intervention, containing information on time spent on guided support, time between finished exercises that were performed by participants and a social worker’s feedback, time spent on peer-to-peer coaching and supervision of sessions, and satisfaction with the intervention.

#### Working mechanism outcomes

The working mechanism, i.e. the psychological mechanisms that might mediate or explain whether the intervention is effective in subgroups of patients, is based on different hypotheses about participants’ perceptions and beliefs about fatigue, and will be measured with the 7-item Brief Illness Perceptions Questionnaire (BIPQ) [51]; the 6-item Self-efficacy scale (SE-scale) to assess how fatigue is handled [52] and the 10-item Fatigue Catastrophizing Scale (FCS) to assess negative cognitions towards fatigue [53]. Additionally, patient characteristics, such as gender and severity of vision loss might moderate the effect. See Additional file 1 for an overview of all measurement instruments and properties.

#### Serious adverse events

The research team will report all study-related SAEs to the accredited medical ethics committee within 7 days of first knowledge for SAEs that result in death or life-threatening. All other study-related SAEs will be reported within a period of maximum of 15 days. All study-related SAEs will be followed until a stable situation has been reached and the patients’ GP is contacted or until they have eased.

### Statistical analysis

#### Clinical effectiveness analysis

Clinical effectiveness will be analyzed using two-sided tests with a significance level of P< 0.05. Linear mixed modeling will be used to compare the change in primary and secondary clinical outcome measures over time between both trial arms, based on the intention-to-treat principle (i.e. all data will be included independent of treatment completion). The models will be adjusted for confounders if necessary. If feasible, questionnaires with latent constructs will be analyzed using item response theory (IRT) models. Effectiveness analyses will be performed in IBM SPSS version 26.0 (IBM SPSS Inc., Chicago, IL) and IRT in R, Version 3.5.2. All other descriptive analyses, e.g., concerning non-response, loss to follow-up, comparisons of patient characteristics between intervention and control group, and process evaluation will be analyzed in SPSS version 25 (IBM SPSS Inc., Chicago, IL). To minimize missing data, participants are contacted to participate in all assessments, even if an assessment has been missed.

#### Cost-effectiveness and cost-utility analysis

Cost-effectiveness analysis (CEA) and cost-utility analysis (CUA) will be performed from a healthcare and societal perspective. Missing cost and effect data will be imputed using multiple imputation techniques according to the Multivariate Imputation by Chained Equations (MICE) algorithm [54]. The results of the imputed datasets will be pooled using Rubin’s rules [55]. Bias-corrected and accelerated bootstrapping with 5000 replications will be used to calculate 95% confidence intervals around the mean difference in total costs between the two groups for both perspectives. Incremental cost-effectiveness ratios (ICERs) will be computed. Bootstrapping will be used to estimate the uncertainty surrounding the ICERs, which will be plotted graphically on cost-effectiveness planes. Cost-effectiveness acceptability curves will also be estimated. Findings will be integrated with published reports and literature to extrapolate the findings to a national level.

#### Budget impact analysis

Based on the results of the clinical study, the CEA and the CUA, a budget impact analysis (BIA), will be performed to inform decision-makers on the financial consequences of implementing E-nergEYEze into routine practice. Guidelines by the ISPOR Task Force will be used for the BIA, i.e., relevant features of the health care system, access restrictions, anticipated uptake, and the use and effect of current and the new intervention will be taken into account. The BIA will be performed from the healthcare perspective, which includes direct healthcare costs. The study results will be extrapolated, by means of a simple linear model, from a time horizon of 2 years to 5 years, concerning the entire Dutch population. Due to a lack of registration, only an estimated number of people who are visually impaired in the Netherlands can be provided. The extrapolation will assume a constant incidence of fatigue in visually impaired adults. Also, we expect that the detection rate as found in the trial will be stable over time. A factor that is expected to change with time is the uptake of the E-nergEYEze intervention. This factor will be used for scenario analysis in the BIA. For each perspective, we access costs when 10%, 20%, 30%, and 100% of the target group receive E-nergEYEze. These scenarios will be compared with the baseline scenario, reflecting current care, where 0% of the target group is offered E-nergEYEze. Sensitivity analysis will be performed on relevant parameters such as the uptake of E-nergEYEze and unit costs. The source of the unit prices will vary with the perspective. Also, future costs will be indexed and not discounted. The precision of costs will be in accordance with the described perspectives. All analysis will be performed in STATA.

#### Structural equation modeling

Separate path models will be developed to identify the determinants of fatigue severity within a structural equation modeling framework (direct and indirect associations). Mediation will be investigated by inspection of the estimated direct, indirect, and total effects within the path models. Depending on the outcomes of the separate models, each new model will be analyzed by comparing it to the former model using model-fit indices (maximum likelihood estimation), i.e., RMSEA, SRMR, CFI, and TLI. Finally, one structural equation model explaining the working mechanism of the intervention on fatigue will be presented. Analyses will be performed with Mplus version Analyses will be performed with Mplus version 7.4 [55].

### Data management

Patient data will be encrypted by an individual code (from 1000 to 3000), which can be linked to the patient with a separately saved “key file.” Only the research team has access to this key file and it will be deleted after the study has ended. Data will be entered into Castor (data entry software) and converted into the statistical software packages used in this trial. The computer network of Amsterdam University Medical Centers will be used to store all data with a password. The registration forms and signed informed consent forms will be kept in a locked cabinet in a locked room, that is only accessible to the research team. Data will be stored for 15 years, after which it will be destroyed. Minddistrict, the Internet Portal Supplier, will encrypt and securely store all data of patients, i.e. assignments and communication with social workers.

## Discussion

Having vision loss is known to compromise the quality of life and puts people at risk for detrimental health outcomes [3, 4, 36, 56]. Severe fatigue on top of visual impairment is associated with incremental societal costs that largely determine the economic burden of low vision or blindness through loss in work participation and increased healthcare utilization [1]. Currently, there is little evidence that interventions offered within low vision service organizations are beneficial for health- and vision-related quality of life. The proposed intervention contributes to the evidence base of potentially feasible interventions to reduce the important health-related consequences of vision loss [57].

This study is innovative since (1) it focuses on eHealth in people with visual impairment which has only marginally been attempted in previous studies, (2) it focuses on vision-related fatigue which is a significant problem in people with visual impairment for which no evidence-based interventions exist, and (3) it is based on a well-designed RCT.

The blended-care character of E-nergEYEze may be a promising approach for both patient and professional. The intervention has been designed specifically for adults with visual impairment by a group of experts. The tailor-made approach to address specific symptoms of fatigue for visually impaired adults will promote its relevance. E-nergEYEze aims to be accessible, independent of time and place, it stimulates patient empowerment and it may increase treatment capacity by professionals. Taking into account the professionals’ and patients’ perspective, thereby stressing its human-centered design, fits the needs and preferences of all end-users.

The usability and feasibility study will provide insight in the accessibility and feasibility of E-nergEYEze. The RCT will determine if a blended vision-specific eHealth intervention based on cognitive behavioral and self-management leads to a significant reduction of fatigue severity. The pragmatic design of the study allows the intervention to be similar to daily clinical practice, which enhances the generalizability of the results and contributes to its future implementation. These strengths provide opportunities for a cost-effective intervention.

There are possible limitations to mention. First, participants and professionals cannot be masked due to the nature of the study. Results might be overestimated by the intervention group, for example, because a participant who receives E-nergEYEze may be more motivated to positively appraise fatigue symptoms than someone in the control group. This could lead to information bias. Nevertheless, the pragmatic design allows for a good representation of future clinical practice. Second, E-nergEYEze will be available online, for which an internet connection is required. Although it seems counterintuitive, people with a visual impairment frequently use internet applications [58]. Since having access and skills to follow the online intervention are necessary, low vision services will provide intervention-specific assistance at home or at the center to address these needs. Third, the control group will receive care as usual, but the expected effects may have been greater if there was an inactive comparator, such as a control group receiving no care or being on a waiting list. Fourth, the mean difference of 4.0 on the CIS-FS is smaller than the clinical relevant difference of 6.0 in previous studies. This smaller difference was chosen as a mean difference of 6.0 was not achieved after one year in several studies [10, 12, 19, 24]. To accommodate this choice, this study will have a larger sample size. Finally, the follow-up period could be considered short, since the intervention lasts 5 months with a 12-months follow-up from baseline. It would be valuable to determine if expected positive effects of E-nergEYEze are sustained over a longer period of time; however, there is no time or resources to investigate a longer maintenance effect and most of the studies on low vision rehabilitation have a follow-up period of less than 12 months [57].

This intervention has been developed with all important stakeholders of low vision services in the Netherlands to secure a pragmatic design and future implementation success of potentially feasible eHealth interventions. If E-nergEYEze shows that eHealth is an effective treatment for severe fatigue in visually impaired adults, with respect to accessibility and content, the intervention and its results could fulfill the gap in knowledge, skills, and treatment options in low vision services in the Netherlands.

## Trial status

Protocol version number 7, date 04-03-2020. Recruitment began on 6 October 2020 and will be completed in April 2022.

## Supplementary Information

**Additional file 1.** Overview measurements [59, 60].

## Data Availability

The datasets analyzed during the current study are available from the corresponding author on reasonable request.

## Abbreviations

CBT: Cognitive behavioral therapy
SM: Self-management
MS: Multiple sclerosis
RCT: Randomized controlled trial
CIS-FS: Checklist Individual Strength – subscale Fatigue Severity
ICT: Information and communication technology
SAE: Serious adverse event
T0: Baseline assessment
MFIS: Modified Fatigue Impact Scale
AVL: Adaptation to Vision Loss
PHQ-9: Patient Health Questionnaire
HADS-A: Hospital Anxiety Depression Scale – subscale Anxiety
IVI: Impact of Visual Impairment
WRFQ: Work Role Functioning Questionnaire
WAS: Work Ability Score
NFR: Need for Recovery
HSDQ: Holland Sleep Disorders Questionnaire
PLE: Past Life Events
EQ-5D-5L: EuroQol-5 Dimensions with 5 Levels
iMCQ: Medical Costs Questionnaire
iPCQ: iMTA Productivity Cost Questionnaire
CCTS-SR: Competencies of Cognitive Therapy Scale – Self Report
MH: Mental Healthcare
BIPQ: Brief Illness Perceptions Questionnaire
SE scale: Self-Efficacy scale
FCS: Fatigue Catastrophizing Scale
IRT: Item Response Theory
CEA: Cost-Effectiveness Analysis
CUA: Cost-Utility Analysis
MICE: Multiple Imputation by Chained Equations
ICERs: Incremental Cost-Effectiveness Ratios
BIA: Budget Impact Analysis
WMO: Medical Research Involving Human Subjects Act
GDPR: EU General Data Protection Regulation
SPIRIT: Standard Protocol Items Recommendations for Interventional Trials
