# Supplementary material for: E-nergEYEze, a vision-specific eHealth intervention based on cognitive behavioral therapy and self-management to reduce fatigue in adults with visual impairment: study protocol for a randomized controlled trial
Source: Trials. 2021 Dec 28;22:966. doi: 10.1186/s13063-021-05935-w (PMC8715593; doi:10.1186/s13063-021-05935-w)
Supplement: Supplementary file 1 — Additional file 1. Overview measurements [59, 60]. [file 13063_2021_5935_MOESM1_ESM.docx]

| Overview of all measurement instruments and properties | | | | | | |
| --- | --- | --- | --- | --- | --- | --- |
| Questionnaire | **Construct of assessment** | **items** | **Response options** | **Scoring** | **Cronbach’s alpha** | **Measurement time-points** |
| General questionnaire | Demographics and comorbidities | 17 items | Multiple response options | - | Not applicable | T0 |
| Primary outcome |  |  |  |  |  |  |
| Checklist Individual Strength, subscale Fatigue Severity  (CIS-FS scale) (30,33) | Fatigue severity | FS: 8 items  (total 20) | 7-point Likert scale:  agree(1) - disagree(7) | Range CIS-FS:  8 – 57  >=35 indicating  severe fatigue | Excellent  (α = 0.94) | T0, T2, T4 |
| Secondary outcomes |  |  |  |  |  |  |
| *Clinical effectiveness:* |  |  |  |  |  |  |
| Checklist Individual Strength (CIS) (30,33) | Additional subscales: concentration, motivation and activity | Total  20 items | 7-point Likert scale:  agree(1) - disagree(7) | Range total scale:  20-140 | Total scale:  Excellent  (α = 0.95) | T0, T2, T4 |
| Modified Fatigue Impact Scale (MFIS) (34) | Impact of fatigue | 21 items | 5-point Likert scale:  Never(0), rarely, sometimes, often, almost always(4) | Range: 0 – 84  Higher number indicating greater fatigue | Excellent  (α = 0.92) | T0, T2, T4 |
| Adaptation to vision loss  (AVL-9)^a^ (35,36) | Adaptation and acceptance of the disability in relation to oneself or towards others | 9 items | 4-point Likert scale:  Strongly disagree, fairly disagree, fairly agree, strongly agree | Range: 0 – 27  ﻿Higher score indicating better adaptation | Good  (α = 0.8 in patients >65 years; α = 0.7 in <65 years)^b^ | T0, T2, T4 |
| Patient Health Questionnaire  (PHQ-9) (37) | Symptoms of depression | 9 items | 4-point Likert scale:  Not at all, several days, more than half of the days, almost every day | Range 0 – 27  ﻿﻿>=10 suggest risks of moderate to severe depression | Good  (α >= 0.7)^c^ | T0, T2, T4 |
| Hospital Anxiety Depression scale subscale Anxiety  (HADS-A) (38) | Symptoms of anxiety | 7 items | 4-point Likert scale:  Rarely or never, sometimes, often, (almost) always | Range 7 - 28  >= 8 indicating caseness of anxiety disorders | Good  (α = 0.83) mean | T0, T2, T4 |
| Impact of Visual Impairment (IVI) (39,40) | Vision-related quality of life | 28 items | 4-point Likert scale:  not at all, a little, moderately, a lot, (not applicable) | Higher values indicating vision-related quality of life | Good  (α = 0.90) | T0, T2, T4 |
| Work Role Functioning Questionnaire (WRFQ) (41) | Work functioning | 27 items | 5-point Likert scale:  all the time (100%), most of the time, half of the time (50%), some of the time, none of the time (0%) and does not apply to my job | Range 0 – 100  Higher scores indicating better work role functioning | Consistent structural validity (59) | T0, T2, T4 |
| Work ability score (WAS) (42) | Work participation | 1 item | 10-point Likert scale:  not able to work(1) - work ability in your best period(10) | Range 1 – 10  Higher scores indicating higher work ability | Not applicable | T0, T2, T4 |
| Need for Recovery (NFR) (43) | Perception and assessment of work | 11 items | No, yes | Range 0 – 100  Higher score indicating a higher degree of need for recovery after work. | Good  (α = 0.88) | T0, T2, T4 |
| Holland Sleep Disorders Questionnaire (HSDQ) (44) | Circadian rhythm sleep disorders, insomnia and hypersomnia | 32 items | 5-point Likert scale:  not at all, usually not, sometimes, usually, completely | Range: 6 – 130  > 2.02 (average) indicating general sleep disorder | Excellent  (α = 0.90) | T0, T2, T4 |
| Past Life Events (PLE) (45) | Life events that happened in the past year | 13 items | Yes, no | - | Not applicable | T4 |
| *Cost-effectiveness:* |  |  |  |  |  |  |
| iMTA Medical Costs Questionnaire (iMCQ) | Health care utilization and medication use | 35 items | Multiple answer options | - | Not applicable | T0, T1, T2,  T3, T4 |
| iMTA Productivity Cost Questionnaire (iPCQ) (46) | Absenteeism and presenteeism from paid and unpaid work | 12 items | Multiple answer options | - | Many items have been validated | T0, T1, T2,  T3, T4 |
| Euroqol-5 Dimensions with 5 levels (EQ-5D-5L) (48) | Health-related quality of life | 6 items | 5-point Likert scale:  not present – constantly present | A 3-point scale score results in a 5-digit index, which is converted into a total score | Acceptable  (α = 0.69) (60) | T0, T2, T4 |
| *Process evaluation:* |  |  |  |  |  |  |
| Competencies of Cognitive Therapy Scale-Self Report  (CCTS-SR) (49) | Cognitive therapy skills | 29 items | 7-point Likert scale:  not at all(1) - completely(7) | Range 29 – 203  Higher score indicating more ﻿cognitive therapy skills | Excellent  (intake α = 0.93, post-treatment  α = 0.97) | T0, T2 |
| Dutch Mental Healthcare (MH) thermometer (50) | Patient satisfaction | 17 items | Multiple answer options | - | Not applicable | T2^d^ |
| Therapist satisfaction and adherence | Recall and compliance | 11 items | Multiple answer options | - | Not applicable | T2^d^ |
| *Working mechanism:* |  |  |  |  |  |  |
| Brief Illness Perceptions Questionnaire (BIPQ) (51) | Perceptions and beliefs about fatigue | 9 items | 10-point Likert scale:  no at all(1) - very much(10) | Range 0 – 90  Higher score indicating more negative perceptions | Good predictive validity | T0, T2, T4 |
| Self-efficacy scale  (SE scale) (52) | Self-efficacy | 6 items | 5-point Likert scale:  strongly disagree, disagree, neutral, agree, strongly agree | Higher score indicating higher perceived general self-efficacy | Good  (α = 0.79  -0.88) | T0, T2, T4 |
| Fatigue Catastrophizing Scale (FCS) (53) | Negative cognitions towards fatigue | 10 items | 5-point Likert scale:  never, sometimes, regularly, mostly, always | Range 10 – 50  ﻿Higher scores are indicative for more fatigue catastrophizing | Good  (α = 0.85) | T0, T2, T4 |

^a^ AVL-9 was adapted from the AVL-12 after IRT analysis (Horowitz)

^b^ Unpublished data

^c^based on person separation reliability (PSR)

^d^intervention group only
